# Common Laboratory Mice Are Susceptible to Infection with the SARS-CoV-2 Beta Variant

**DOI:** 10.3390/v13112263

**Published:** 2021-11-11

**Authors:** Ravi Kant, Lauri Kareinen, Teemu Smura, Tobias L. Freitag, Sawan Kumar Jha, Kari Alitalo, Seppo Meri, Tarja Sironen, Kalle Saksela, Tomas Strandin, Anja Kipar, Olli Vapalahti

**Affiliations:** 1 Zoonosis Unit, Department of Virology, Medicum, University of Helsinki, 00290 Helsinki, Finland; lauri.kareinen@helsinki.fi (L.K.); teemu.smura@helsinki.fi (T.S.); tarja.sironen@helsinki.fi (T.S.); kalle.saksela@helsinki.fi (K.S.); olli.vapalahti@helsinki.fi (O.V.); 2 Department of Basic Veterinary Sciences, Faculty of Veterinary Medicine, University of Helsinki, 00790 Helsinki, Finland; anja.kipar@uzh.ch; 3 Research Programs Unit, Immunobiology, University of Helsinki, 00290 Helsinki, Finland; tobias.freitag@helsinki.fi; 4 Translational Cancer Medicine Program, Faculty of Medicine and Helsinki Institute of Life Science, University of Helsinki, 00290 Helsinki, Finland; jha.sawankumar@helsinki.fi (S.K.J.); kari.alitalo@helsinki.fi (K.A.); 5 Department of Bacteriology and Immunology and Translational Immunology Research Program, University of Helsinki, 00290 Helsinki, Finland; seppo.meri@helsinki.fi; 6 Laboratory for Animal Model Pathology, Institute of Veterinary Pathology, Vetsuisse Faculty, University of Zurich, 8057 Zurich, Switzerland; 7 Department of Infection Biology & Microbiomes, Institute of Infection, Veterinary and Ecological Sciences, University of Liverpool, Liverpool L3 3RF, UK; 8 HUS Diagnostic Center, HUSLAB, Clinical Microbiology, Helsinki University Hospital, 00290 Helsinki, Finland

**Keywords:** SARS-CoV2, COVID-19, SARS-CoV-2 variants, SARS-CoV-2 beta variants, infections, laboratory mice, common laboratory mice

## Abstract

Small animal models are of crucial importance for assessing COVID-19 countermeasures. Common laboratory mice would be well-suited for this purpose but are not susceptible to infection with wild-type SARS-CoV-2. However, the development of mouse-adapted virus strains has revealed key mutations in the SARS-CoV-2 spike protein that increase infectivity, and interestingly, many of these mutations are also present in naturally occurring SARS-CoV-2 variants of concern. This suggests that these variants might have the ability to infect common laboratory mice. Herein we show that the SARS-CoV-2 beta variant attains infectibility to BALB/c mice and causes pulmonary changes within 2–3 days post infection, consistent with results seen in other murine models of COVID-19, at a reasonable virus dose (2 × 10^5^ PFU). The findings suggest that common laboratory mice can serve as the animal model of choice for testing the effectiveness of antiviral drugs and vaccines against SARS-CoV-2.

## 1. Introduction

The COVID-19 pandemic has been affecting the world since the emergence of SARS-CoV-2 in late 2019. Effective viral spread among the human population is potentiated by the emergence of highly transmittable variants of concern (VOCs) some of which can evade previously acquired immunity and cause more severe disease than the “wild-type” (wt) strains originating from Wuhan [1,2]. According to the nomenclature declared by the World Health Organization (WHO), the VOCs include alpha (B.1.1.7 by Pango nomenclature), beta (B.1.351), gamma (P.1) and delta (B.1.617.2) [3].

The rapid development of vaccines and studies of antiviral drugs against SARS-CoV-2 are of prime importance and require animal models that mimic human SARS-CoV-2 infection and the associated disease. Several animal species, such as Syrian hamsters and nonhuman primates, are particularly suitable for in vivo approaches [4]. In contrast, common laboratory mice were shown to be refractory to wt virus infection due to mouse-specific differences in the SARS-CoV-2 entry receptor, the angiotensin converting enzyme (ACE)-2 [5,6]. However, using the mouse as the model species would provide substantial benefits, as it would allow application of the large toolbox for studies on host genetics and immune responses developed over decades by the scientific community. Introduction of human ACE-2 (hACE-2) into mice partially overcomes this drawback but these models are not adequate for all questions as they do not faithfully mimic the natural expression pattern of the ACE-2 receptor [4].

Instead of mutating the host, SARS-CoV-2 infection models in mice have also been achieved by mutating the virus by serial passaging through mouse lungs. By these means a few key residues that improve the affinity of the SARS-CoV-2 spike S1 protein to mouse ACE-2 (mACE-2) and increase infectivity of SARS-CoV-2 in standard BALB/c mice have been observed [7,8]. Interestingly, some of these mutations appear also in circulating VOCs, such as the N501Y mutation present in alpha, beta and gamma. This suggests that isolates of these VOCs would likely possess improved infectivity over wt SARS-CoV-2 in mice. In order to test this hypothesis, we challenged BALB/C mice intranasally with the beta variant and wt SARS-CoV-2 and assessed infectivity as well as dissemination and pathological effects within infected animals.

## 2. Materials and Methods

### 2.1. Animals

A total of 30 male and female BALB/c mice (Envigo, Indianapolis, IN, USA) were transported to the University of Helsinki biosafety level-3 (BSL-3) facility and acclimatized to individually ventilated biocontainment cages (ISOcage; Scanbur, Karl Sloanestran, Denmark) for seven days with ad libitum water and food (rodent pellets). For subsequent experimental infection, the mice were placed under isoflurane anaesthesia and inoculated intranasally with 20 µL of virus dilution or PBS (mock-infected control; one animal). The animals were held in an upright position for a few seconds to allow the liquid to flush downwards in the nasal cavity. All mice were weighed on a daily basis. Their wellbeing was further monitored carefully for signs of illness (e.g., changes in posture or behaviour, rough coat, apathy, ataxia). Euthanasia was performed under terminal isoflurane anaesthesia with cervical dislocation. Experimental procedures were approved by the Animal Experimental Board of Finland (license number ESAVI/28687/2020).

### 2.2. Virus Isolation and Sequencing

The wt/D614G strain and beta variant SARS-CoV-2 viruses were isolated using transmembrane serine protease 2 (TMPRSS2)-transduced Vero E6 cells from SARS-CoV-2 infected patient nasopharyngeal samples as described in [1,9]. RNA from isolated virus preparations or lung tissues were used for sequencing with the Illumina platform in-house in the Department of Virology, University of Helsinki [9]. The wt and beta variant sequences have been deposited in the NCBI GenBank database under accession numbers MZ962407 and MW717678, respectively, and are described in detail in [1].

### 2.3. Infection Experiments

Groups of 9-, 12- or 14-week-old male and female BALB/c mice were intranasally inoculated with either the beta variant (*n* = 22) or wt strain (*n* = 7) of SARS-CoV-2, or mock-infected with PBS (*n* = 1) [1]. Eleven animals received a dose of 2 × 10^5^ plaque-forming units (PFU); five of these (all female and 9 weeks old) were euthanised at 2 days post infection (dpi), the remaining six (all male and 12 weeks old) at 3 dpi. Five female animals received wt virus at the same dose and were euthanized at 3 dpi. In addition, each of the four 14-week-old male mice received the beta variant at a dose of 6 × 10^4^ PFU and 6 × 10^3^ PFU, respectively, and two male mice received wt virus (6 × 10^4^ PFU); all were euthanized at 2 dpi. Another three 12-week-old female animals were challenged with 6 × 10^4^ PFU beta SARS-CoV-2 and were euthanized at 4 dpi. One 14-week-old male mouse was mock infected with PBS and culled after two days (negative control). All animals were dissected immediately after death; the right lungs were collected and frozen for virological examinations, whereas the left lung, remaining thoracic organs and heads were fixed in 10% buffered formalin for 48 h and stored in 70% ethanol for histological and immunohistochemical examinations.

### 2.4. Virus Titration

In order to measure viral titres in mouse lungs, the frozen right lungs (high dose, 2 and 3 dpi for beta and wt, respectively; *n* = 5 both) were first homogenized with mortar and pestle in 1 mL of cell growth medium (Minimum essential medium supplemented (Sigma-Aldrich, St. Louis, MO, USA) with 2% foetal calf serum, 2 mM of L-glutamine, 100 IU/mL of penicillin and 100 μg/mL of streptomycin) on dry ice, which after 10-fold dilutions of the cleared (by centrifugation at 500× *g* for 5 min) homogenate was applied on Vero E6 cell cultures. After two days of culture, cells were fixed with 4% paraformaldehyde for 10 min, blocked, permeabilized (3% BSA; 0.1% Triton-X100 in PBS) for 10 min and stained for SARS-CoV-2 receptor binding domain (RBD) by a specific rabbit polyclonal antibody [10] followed by AF488-conjugated secondary antibody (ThermoScientific, Waltham, MA, USA). Nuclei were stained with Hoechst 33420 (Sigma Aldrich, St. Louis, MO, USA). After imaging with the PerkinElmer Opera Phenix spinning disk confocal microscope using a 20× water-immersion objective (NA 1.0), the quantification of RBD-positive cells was conducted with the Harmony software (PerkinElmer, Waltham, MA, USA) by using a supervised linear classifier. The viral titres were reported as the minimal infectious dose resulting in at least 50% infection in tissue culture (TCID50) per whole lung.

### 2.5. RNA Isolation and RT-qPCR

RNA was extracted from lung samples using Trizol (Thermo Scientific) according to the manufacturers’ instructions. Isolated RNA was directly subjected to one-step RT-qPCR analysis based on a previously described protocol for RdRp [11] and for E and subE genes [12] with TaqMan fast virus 1-step master mix (Thermo Scientific) using AriaMx instrumentation (Agilent, Santa Clara, CA, USA). The actin RT-qPCR is described in [13] (Table 1).

### 2.6. Histology and Immunohistochemistry

The left lung, remaining thoracic organs and heads were trimmed for histological examination and routinely paraffin-wax embedded. The heads of the animals that had received beta SARS-CoV-2 and wt virus at a dose of 2 × 10^5^ PFU were sawn longitudinally in the midline using a diamond saw (Exakt 300; Exakt, Oklahoma, OK, USA). In mice that had received lower virus doses and in the mock-infected control mouse, coronal sections (1–2 mm) of the nose were prepared using the saw. Heads were gently decalcified in RDF (Biosystems, Barcelona, Spain) for 5 days at room temperature (RT) and on a shaker, then paraffin-wax embedded. Consecutive sections (3–5 µm) were prepared from lungs and heads and routinely stained with haematoxylin–eosin (HE) or subjected to immunohistochemistry (IHC) for the detection of SARS-CoV-2 antigens, as previously described [13]. IHC was performed in an autostainer (Agilent) using a rabbit polyclonal anti-SARS-CoV nucleoprotein antibody (200-402-A50; Rockland Immunochemicals, Limerick, PA, USA) and the horseradish peroxidase (HRP) method. Briefly, sections were deparaffinized and rehydrated through graded alcohol. Antigen retrieval was achieved by 20 min incubation in citrate buffer (pH 6.0) at 98 °C in a pressure cooker. This was followed by incubation with the primary antibody (diluted 1:3000 in dilution buffer; Dako) overnight at 4 °C, a 10 min incubation at RT with peroxidase blocking buffer (Agilent) and a 30 min incubation at RT with Envision + System HRP Rabbit (Agilent). The reaction was visualized with diaminobenzidine (DAB; Dako) for 10 min at RT. After counterstaining with haematoxylin for 2 s, sections were dehydrated and placed on a coverslip with Tissue-Tek Film (Sysmex, Kobe, Japan).

## 3. Results

### 3.1. BALB/c Mice Develop Pulmonary Infection, Alveolar Damage and a Mild Inflammatory Response within 3 Days of Intranasal Challenge with the Beta Variant of SARS-CoV-2

**Figure 1:**
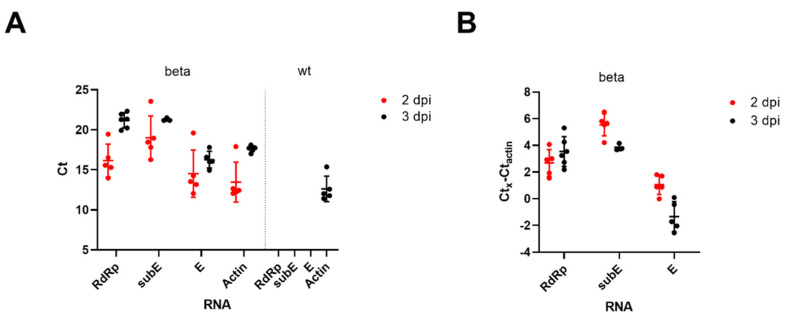
BALB/c mice were intranasally inoculated with 2 × 10^5^ PFU of the beta variant or wt SARS-CoV-2 and euthanized at 2 or 3 days post inoculation (dpi) (*n* = 5 female for beta at 2 dpi and for wt at 3 dpi, *n* = 6 male for beta at 3 dpi), followed by RT-qPCR analysis of RNA isolated from the lungs. (**A**) The Ct values for genomic RdRp and E genes as well as subgenomic E gene (subE) are shown for both beta and wt infections. Actin mRNA levels served as an internal control target. (**B**) The normalized Ct values for RdRp, E and subE at 2 and 3 days post inoculation with beta variant. Normalization was achieved by subtracting actin mRNA Ct values from target Cts. Means ± standard deviation are indicated.

We intranasally inoculated 9- and 12-week-old BALB/c mice with 2 × 10^5^ PFU of beta or wt SARS-CoV-2 (Supplementary Table S1) and monitored the animals clinically until euthanasia at 2 and 3 dpi. None of the mice showed weight loss or any clinical signs during the study period. We assessed virus replication in the lungs by RT-qPCR and detected high amounts of SARS-CoV-2 RNA in mice infected with the beta variant, as indicated by low Ct values (Figure 1) for the RNA-dependent RNA polymerase (RdRp) and envelope (E) genes, as well as the subgenomic E gene (subE; replication intermediate [12]). In mice infected with wt SARS-CoV-2 and the mock-infected control animal, none of the viral RNA targets were detectable in the lungs, as shown previously [14].

**Figure 2:**
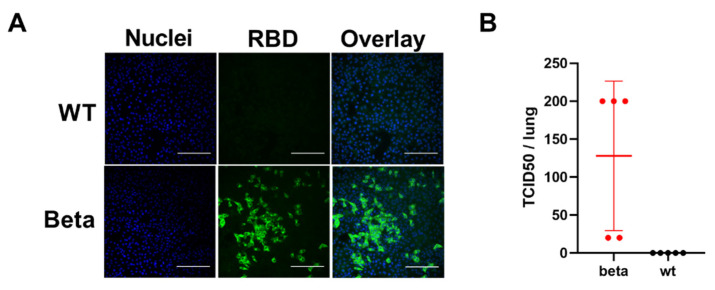
Viral titres in BALB/c mouse lungs after SARS-CoV-2 infection, determined in Vero E6 cells incubated for 2 days with lung homogenate of wt- or beta variant-infected BALB/c mice (2 and 3 dpi for beta and wt, respectively; *n* = 5 both). (**A**) Immunofluorescence image. Viral infection of cells was detected by a RBD-specific rabbit polyclonal Ab, followed by an AF488-conjugated secondary Ab (green). Nuclei were stained with Hoechst 33420 (blue). Bar = 100 µm. (**B**) The TCID50 in lung tissues was calculated as assessed by immunofluorescence.

Virus isolation in cell culture confirmed the presence of infectious virus in the lungs of mice challenged with the SARS-CoV-2 beta variant (Figure 2). No new viral mutations were detected in the lungs of infected mice when compared to those inoculated with the SARS-CoV-2 beta variant, determined by SARS-CoV-2 whole-genome NGS of RNA isolated from lung specimens.

**Figure 3:**
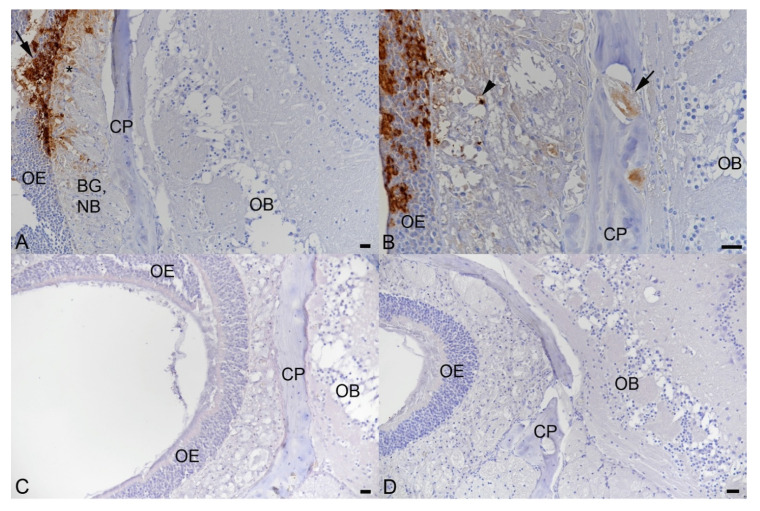
SARS-CoV-2 NP expression in the nose of BALB-C mice after intranasal infection with 2 × 10^5^ PFU of beta variant (**A**,**B**) and wt SARS-CoV-2 (**C**). Olfactory epithelium (OE), bony cribriform plate (CP) and olfactory bulb (OB), midline longitudinal section, head. (**A**). Female animal infected with beta SARS-CoV-2 and euthanized at 2 dpi. Strong, patchy, viral antigen expression in olfactory epithelial cells (arrow) and a few cells (*) in the underlying layer containing Bowman’s glands (BG) and nerve bundles (NB). There is no evidence of viral antigen expression in the olfactory bulb. (**B**). Male animal infected with beta SARS-CoV-2 and euthanized at 3 dpi. Strong, patchy, viral antigen expression in olfactory epithelial cells (OE) and a few cells (arrowhead) in the underlying layer containing Bowman’s glands and nerve bundles. There is also evidence of viral antigen expression in olfactory nerves (arrow) perforating the bony cribriform plate. (**C**). Female animal infected with wt SARS-CoV-2 and euthanized at 3 dpi. There is no evidence of viral antigen expression. (**D**). Mock-infected male animal euthanized at 2 dpi. Again, there is no evidence of viral antigen expression. Bars = 20 µm. Immunohistochemistry, haematoxylin counterstain.

Pathological changes and viral nucleoprotein expression were assessed in nose, lungs and brain. In the nose, immunohistochemistry confirmed replication of beta SARS-CoV-2 in nasal and olfactory epithelium, Bowman’s glands and nerve bundles at both 2 and 3 dpi (Figure 3A,B); at 3 dpi, viral antigen was also detected in occasional olfactory nerves (Figure 3A,B). This was associated with the presence of moderate numbers of sloughed-off degenerate nasal and olfactory epithelial cells and cell-free viral antigen in the nasal cavity. There was no evidence of viral antigen expression in the olfactory bulb and the brain. In wt-infected animals, examined at 3 dpi, and in the mock-infected mouse, viral antigen was not detected in these locations (Figure 3C).

**Figure 4:**
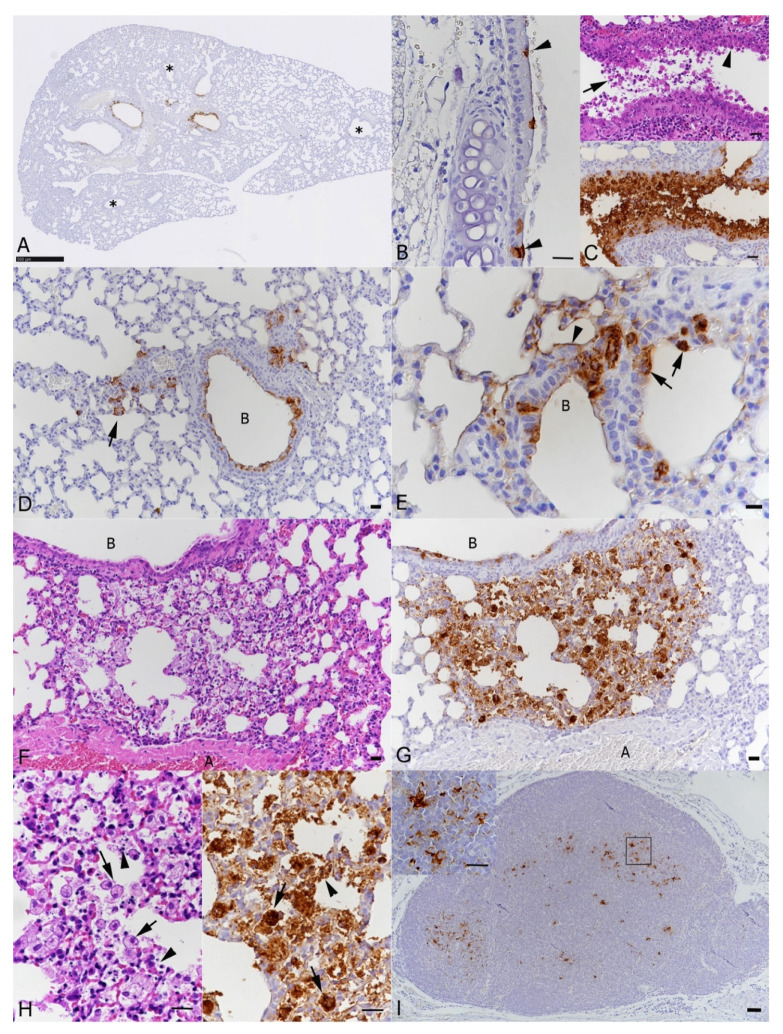
Histopathological changes and SARS-CoV-2 NP expression in airways and lungs of BALB-C mice after intranasal infection with 2 × 10^5^ PFU of beta variant. (**A**–**E**). Female animal euthanized at 2 dpi. (**A**). Left lung lobe, longitudinal section, overview. Viral antigen expression in individual to almost all epithelial cells in several bronchioles. Other bronchioles are entirely negative (*). Bar = 500 µm. (**B**). Trachea with a few individual ciliated epithelial cells expressing viral antigen (arrowheads). Bar = 10 µm. (**C**). Lung; larger bronchiole with degeneration of epithelial cells (arrowhead) and degenerate cells in the lumen (arrow) and extensive viral antigen expression. There is also a mild peribronchiolar lymphocyte infiltration. Bar = 20 µm. (**D**). Lung with viral antigen expression in bronchiolar epithelial cells and pneumocytes in adjacent alveoli (arrow). B: bronchiole. Bar = 20 µm. (**E**). Bronchioalveolar transition. Viral antigen expression is seen in bronchiolar (B) epithelial cells, type I (arrowhead) and type II (arrows) pneumocytes. Bar = 10 µm. (**F**–**I**). Male animal euthanized at 3 dpi. (**F**,**G**). Lung with focal area of alveolar damage and accumulation of cells in alveolar lumina, with intense viral antigen expression. A: artery; B: bronchiole. Bars = 20 µm. (**H**). Closer view of the area in (**F**) and (**G**), showing degeneration of alveolar epithelial cells (arrowheads) and accumulation of macrophages with cytoplasmic vacuolation and phagocytosed cell debris (arrows), associated with strong viral antigen expression. Bar = 10 µm. (**I**). Bronchial lymph node with viral antigen expression in cells with the morphology of macrophages and dendritic cells (inset). Bar = 20 µm (inset: 10 µm). Haematoxylin stain and immunohistochemistry, haematoxylin counterstain.

Examination of the upper and lower airways, i.e., trachea and bronchi/bronchioles, showed viral antigen expression in intact and occasional degenerate epithelial cells (Figure 4A–G). At 2 dpi, only one animal exhibited viral antigen in alveoli; type I and II pneumocytes of a few alveoli adjacent to infected bronchioles were found to be positive (Figure 4D,E). A second mouse showed some activated type II pneumocytes. With more extensive viral antigen expression and degeneration of bronchial epithelial cells, leukocyte infiltration of the bronchiolar wall was seen; this was associated with viral antigen expression within infiltrating macrophages. There was evidence of mild endothelial cell activation in arteries adjacent to affected bronchioles and mild peribronchial lymphocyte infiltration. At 3 dpi, alveolar viral antigen expression was more widespread and associated with clear evidence of alveolar damage, represented by focal areas in which alveoli were filled with vacuolated macrophages, erythrocytes and degenerate cells, necrosis of pneumocytes was also seen (Figure 4F–H). In affected areas, vessels showed endothelial cell activation and occasional rolling and emigration of leukocytes. The observed changes are consistent with a mild bronchointerstitial pneumonia, are and of the same nature as those observed in K18-hACE2 mice at 3 dpi with a wt strain, though less extensive, with the same target cells but less widespread alveolar viral antigen expression and less tissue damage [15]. Bronchial lymph nodes generally exhibited SARS-CoV-2 antigen in several macrophages and/or dendritic cells (Figure 4I). Detailed information on the results in individual animals is provided in Supplementary Table S1.

In contrast, mice inoculated with wt SARS-CoV-2 and examined at 3 dpi did not show any histological changes and no viral antigen expression in the lungs.

### 3.2. Intranasal Challenge of BALB/c Mice with a Low Dose of the SARS-CoV-2 Beta Variant Leads to Nasal Infection but Inconsistent Pulmonary Infection

**Figure 5:**
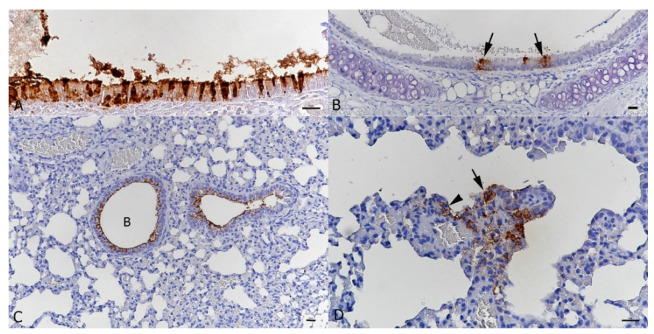
BALB-C mouse, male. Two days post intranasal infection with 6 × 10^3^ PFU of beta variant SARS-CoV-2. (**A**). Nose with focal extensive viral antigen expression in ciliated epithelial cells. Bar = 20 µm. (**B**). Trachea with small patches of positive epithelial cells (arrows). Bar = 20 µm. (**C**). Lung with viral antigen deposition along the luminal surface of the bronchiolar epithelial cells. B: bronchiole. Bar = 20 µm. (**D**). Lung. Small focal area with viral antigen expression in type I (arrowhead) and type II (arrow) alveolar epithelial cells. Bar = 10 µm. Immunohistochemistry, haematoxylin counterstain.

In Syrian hamsters, robust pulmonary SARS-CoV-2 infection can be achieved with intranasal application of relatively low virus doses (in the range of 10^3^ PFU). To study if one could use similarly lower infectious doses of beta variant that results in reproducible infection in the lungs of BALB/c mice, we also attempted intranasal inoculations with 6 × 10^4^ and 6 × 10^3^ PFU and examined the animals at 2 dpi. With both doses of the beta variant, infection of the nasal mucosa was established, as shown by viral antigen expression in patches of respiratory and olfactory epithelial cells (Figure 5A). Occasional leukocyte rolling in vessels beneath the infected epithelium indicated an early inflammatory response. Virus replication in the lungs could not be consistently established with a dose of either 6 × 10^4^ or 6 × 10^3^ PFU, as indicated by the discordant results of immunohistochemistry and PCR (Supplementary Table S1). However, this discordance could potentially be explained because each half of the lungs was processed for either approach. However, the detection of viral antigen in tracheal, bronchiolar and, rarely, alveolar epithelial cells (Figure 5B–D) confirmed that infection could be achieved. In animals inoculated with wt virus at 6 × 10^4^, immunohistochemistry did not provide any evidence of infection in nasal mucosa, airways or lungs. We also asked whether intranasal inoculation with lower doses would require more time to establish efficient infection in the lung. However, the results remained as inconsistent as at 2 dpi with a dose of 6 × 10^4^ PFU and examination at 4 dpi. Again, detailed results for each individual animal are provided in Supplementary Table S1.

## 4. Discussion

The wt SARS-CoV-2 is unable to infect mice due to a receptor mismatch, but accumulating evidence suggests that naturally occurring VOCs might be able to acquire this capability [4,16]. We addressed this hypothesis and showed that BALB/c mice are susceptible to infection with the beta variant and develop pneumonia very similar to K18-hACE2 mice and other species susceptible to SARS-CoV-2, albeit of less intensity [15,17]. Our results suggest that optimal dosing of the beta variant is in the 10^5^ PFU range, to consistently achieve infection of the lungs. For more intense pneumonic changes, an even higher dose might be required. We examined both male and female mice, all of which were young adults (9–14 weeks), and did not find any evidence of differences in susceptibility to infection depending on sex and age; however, due to the small animal numbers and narrow age distribution, we cannot draw any more fundamental conclusions. Additionally, the kinetics of the infection needs to be studied in more detail with further experimentation.

The RBDs of alpha, beta and gamma variants have been shown to bind murine ACE2 with higher affinity than RBD of the wt/D614G strain [18]. This increased binding can be explained by in silico predictions based on comparative structural analysis of different VOC RBDs towards N501Y mutation as key in enhanced mACE2 recognition [19]. The combination of this in silico information with the results of the experimental work from our group and others [20] strongly suggests common mice (*Mus musculus*) as another animal species susceptible to SARS-CoV-2 infection. This indicates a possibility for mice to spread the virus and provide a peridomestic reservoir for SARS-CoV-2 with potential of divergent evolution and transmission (back) to humans. Given the global distribution of common mice and their close association to humans as pets, this might pose a challenge. However, the occurrence of mouse-to-mouse transmission remains to be studied experimentally, as well as whether the now globally dominant delta variant would possess the ability to infect mice. However, the lack of N501Y or other mutations associated with increased mACE2 affinity suggest this would be unlikely [19].

At this stage we did not find immunohistochemical evidence of brain infection. This is in line with several studies in wt-infected K18-hACE2 mice where brain infection was generally not detected prior to 5 dpi [15]. However, we found evidence of viral spread towards the olfactory bulb, as we detected viral antigen in olfactory nerves. This provides initial evidence that the beta variant may enter the brain in a similar fashion as in wt infection of K18-hACE2 mice, via the olfactory bulb [21].

## 5. Conclusions

In this report, we show that unlike wt SARS-CoV-2, the beta variant can infect common BALB/c laboratory mice and causes pulmonary changes consistent with those seen in other murine models of COVID-19, though less severe. Using an intranasal dose of 2 × 10^5^ PFUs, a robust and reproducible airway and lung infection is obtained within 2–3 days post infection. Further studies are needed to (a) better understand the kinetics of infection and ensuing immune responses in BALB/c mice; (b) determine whether/which other variants can infect laboratory mice and the genetic determinants required, and (c) reveal the basis of the acquired ability of the virus to infect laboratory mice, i.e., an increased affinity to mACE2 specifically or to ACE2 across species (including hACE2). While the findings may suggest some potential for mice as peridomestic SARS-CoV-2 reservoir which requires more studies, the main outcome of this study is the option to experimentally approach various COVID-19 countermeasures in an unprecedented scale, using SARS-CoV-2 beta variant infection in laboratory mice and the large genetic and immunological toolbox available for such studies.

## Figures and Tables

**Table 1:** Primer and probe sequences used in the RT-qPCR.

Target		Sequence	Ref.
RdRp	Forward	gtgaratggtcatgtgtggcgg	[11]
	Probe	caggtggaacctcatcaggagatgc	[11]
	Reverse	caratgttaaasacactattagcata	[11]
Subgenomic E	Forward	cgatctcttgtagatctgttctc	[12]
	Probe	acactagccatccttactgcgcttcg	[12]
	Reverse	atattgcagcagtacgcacaca	[12]
Genomic E	Forward	acaggtacgttaatagttaatagcgt	[12]
	Probe	acac-tagccatccttactgcgcttcg	[12]
	Revere	atattgcagcagtacgcacaca	[12]
Beta-actin	Forward	actgccgcatcctcttcct	[13]
	Probe	cctggagaagagctatgagctgcctgatg	[13]
	Reverse	tcgttgccaatggtgatgac	[13]

## Data Availability

Not applicable.

## Supplementary Materials

The following are available online at https://www.mdpi.com/1999-4915/13/11/2263/s1, Figure S1: SARS-CoV-2 nucleoprotein and RNA expression in mice infected with the beta variant with different infectious doses at 2, 3 or 4 days post infection.
