# Supplementary material for: Common Laboratory Mice Are Susceptible to Infection with the SARS-CoV-2 Beta Variant
Source: Viruses. 2021 Nov 11;13(11):2263. doi: 10.3390/v13112263 (PMC8619350; doi:10.3390/v13112263)
Supplement: Supplementary file 1 [file viruses-13-02263-s001.zip › viruses-1430376-supplementary.pdf]

Table S1. SARS-CoV-2 nucleoprotein and RNA expression in mice infected with the beta variant with different infectious doses at 2, 3 or 4 days post infection. **Legend**: Ag – antigen; BG – Bowman's glands; BLN – bronchial lymph node; DC – dendritic cells; EC – epithelial cells; NB – nerve bundles; NE – not examined; neg – negative; OEC – olfactory epithelial cells; pos – positive; REC – respiratory epithelial cells; RdRp PCR: Ct_{RdRp}-Ct_{actin} ≤ 3 = ++, > 3 = +, no Ct = -; subE PCR: Ct_{subE}-Ct_{actin} ≤ 4.5 = ++, > 4.5 = +, no Ct = -; E PCR: Ct_{E}-Ct_{actin} ≤ -1 = ++, -1 ≤ 10 = +, > 10 = -.

**Dose<br>[Animal no]**	**DPI**	**Viral Ag**	**Viral RNA in lung (RT-qPCR)**		
			**RdRp**	**subE**	**E**
**2 x 10^5 PFU<br>[1]**	**2**	**Nose**: individual pos REC/OEC, patches of pos REC/OEC; pos EC in BG<br>**Brain**: neg (incl olfactory bulb)<br>**Trachea**: several individual pos EC<br>**Lung**: bronchus, bronchioles: individual and patches of pos EC, viral Ag covering luminal surface; some adjacent small patches of alveoli with pos type I and II pneumocytes<br>**BLN**: several pos macrophages/DC	++	+	+
**2 x 10^5 PFU**<br>[2]	**2**	**Nose**: individual pos REC/OEC, patches of pos REC<br>**Brain**: neg (incl olfactory bulb)<br>**Trachea**: NE<br>**Lung**: several bronchioles with individual to abundant pos EC, viral Ag covering luminal surface; some individual adjacent pos type I and II pneumocytes<br>**BLN**: NE	+	+	+
**2 x 10^5 PFU**<br>[3]	**2**	**Nose**: some individual pos REC/OEC, patches of pos REC<br>**Brain**: neg (incl olfactory bulb)<br>**Trachea**: several individual pos EC<br>**Lung**: bronchioles with several individual and patches of pos EC; viral Ag covering luminal surface; areas with degenerate EC and leukocyte infiltration with viral Ag also in infiltrating macrophages; patches of alveoli with pos type I and II pneumocytes<br>**BLN**: neg	++	+	+
**2 x 10^5 PFU**<br>[4]	**2**	**Nose**: some individual pos REC/OEC, patches of pos REC<br>**Brain**: neg (incl olfactory bulb)<br>**Trachea**: NE<br>**Lung**: bronchioles with several individual and patches of pos EC and some degenerate pos cells in lumen; viral Ag covering luminal surface; areas with degenerate EC and leukocyte infiltration with viral Ag also in infiltrating macrophages; patches of alveoli with pos type I and II pneumocytes<br>**BLN**: a few pos macrophages/DC	++	+	+
**2 x 10^5 PFU**<br>[5]	**2**	**Nose**: some individual pos REC/OEC, large stretch of pos REC<br>**Brain**: neg (incl olfactory bulb)<br>**Trachea**: a few pos EC<br>**Lung**: most bronchioles with several individual and patches of pos EC; viral Ag covering luminal surface; areas with degenerate EC and leukocyte infiltration with viral Ag also in infiltrating macrophages; patches of alveoli with pos type I and II pneumocytes<br>**BLN**: several pos macrophages/DC	++	++	+
**2 x 10^5 PFU**<br>[1]	**3**	**Nose**: large patches of pos REC/OEC<br>**Brain**: neg (incl olfactory bulb)<br>**Trachea**: rare individual pos EC<br>**Lung**: bronchus and bronchioles with individual and patches of pos EC; abundant viral Ag covering luminal surface; adjacent several patches of alveoli with pos type I and II pneumocytes, sloughed off pos alveolar	++	++	++

		macrophages/type II pneumocytes, and cell free viral Ag in alveolar lumen<br>**BLN**: numerous pos macrophages/DC			
**2 x 10^5 PFU**<br>[2]	**3**	**Nose**: patches of pos REC; large area of pos OEC; some pos cells in BG and NB layer<br>**Brain**: neg (incl olfactory bulb)<br>**Trachea**: rare individual pos EC<br>**Lung**: bronchus and bronchioles with individual and patches of pos EC; abundant viral Ag covering luminal surface; rare small patches of alveoli with pos type I and II pneumocytes, sloughed off pos alveolar macrophages/type II pneumocytes, and cell free viral Ag in alveolar lumen<br>**BLN**: several pos macrophages/DC	+	-	+
**2 x 10^5 PFU**<br>[3]	**3**	**Nose**: large patches of pos REC<br>**Brain**: neg (incl olfactory bulb)<br>**Trachea**: rare individual pos EC<br>**Lung**: bronchus with rare pos EC, bronchioles often with abundant viral Ag covering luminal surface; adjacent a few patches of alveoli with pos type I and II pneumocytes, sloughed off pos alveolar macrophages/type II pneumocytes, and cell free viral Ag in alveolar lumen<br>**BLN**: numerous pos macrophages/DC	+	-	++
**2 x 10^5 PFU**<br>[4]	**3**	**Nose**: large patches of pos REC<br>**Brain**: neg (incl olfactory bulb)<br>**Trachea**: a few individual pos EC<br>**Lung**: bronchus and some bronchioles with few individual and patches of pos EC; abundant viral Ag covering luminal surface; adjacent large patch of alveoli with pos type I and II pneumocytes, sloughed off pos alveolar macrophages/type II pneumocytes, and cell free viral Ag in alveolar lumen<br>**BLN**: several pos macrophages/DC	+	-	++
**2 x 10^5 PFU**<br>[5]	**3**	**Nose**: a few individual pos REC; a few patches of pos REC<br>**Brain**: neg (incl olfactory bulb)<br>**Trachea**: rare individual pos EC<br>**Lung**: bronchus and bronchioles with individual and patches of pos EC; abundant viral Ag covering luminal surface; adjacent several patches of alveoli with pos type I and II pneumocytes, sloughed off pos alveolar macrophages/type II pneuomcytes, and cell free viral Ag in alveolar lumen<br>**BLN**: numerous pos macrophages/DC	++	++	-
**2 x 10^5 PFU**<br>[6]	**3**	**Nose**: patches of pos REC; large area of pos OEC; some pos cells in BG and NB layer and olfactory nerves<br>**Brain**: neg (incl olfactory bulb)<br>**Trachea**: rare individual pos EC, a few pos degenerate cells in lumen<br>**Lung**: bronchus with rare pos EC, abundant viral Ag covering luminal surface; adjacent a few patches of alveoli with pos type I and II pneumocytes, sloughed off pos alveolar macrophages/type II pneumocytes, and cell free viral Ag in alveolar lumen<br>**BLN**: numerous pos macrophages/DC	+	++	+
**6 x 10^4 PFU**<br>[1]	**2**	**Nose**: several patches of and individual pos REC and OEC; some viral Ag attached to epithelial surface<br>**Olfactory bulb**: neg<br>**Trachea**: patches of pos EC<br>**Lung**: abundant viral Ag deposition covering luminal surface of several bronchioles	-	-	-

**6 x 10^4 PFU**<br>[2]	**2**	**Nose**: some patches of and several individual pos REC<br>**Olfactory bulb**: neg<br>**Trachea**: one patch of pos EC<br>**Lung**: neg	-	-	-
**6 x 10^4 PFU**<br>[3]	**2**	**Nose**: abundant patches of and individual pos REC; some cell free viral Ag in mucus<br>**Olfactory bulb**: neg<br>**Trachea**: neg<br>**Lung**: neg	-	-	-
**6 x 10^4 PFU**<br>[4]	**2**	**Nose**: abundant patches of and individual pos REC, occasional pos OEC; cell free viral Ag in mucus<br>**Trachea**: neg<br>**Lung**: neg	-	-	-
**6 x 10^4 PFU**<br>[1]	**4**	**Nose**: individual and groups of pos REC and OEC; some cell free viral Ag in mucus<br>**Olfactory bulb**: neg<br>**Trachea**: neg<br>**Lung**: neg	-	-	-
**6 x 10^4 PFU**<br>[2]	**4**	**Nose**: individual and groups of pos REC, a few pos OEC<br>**Lung**: one bronchiole and one bronchio-alveolar transition with a few pos EC<br>**BLN**: several pos macrophages/DC	-	-	-
**6 x 10^4 PFU**<br>[3]	**4**	**Nose**: numerous pos OEC; cell free viral Ag in mucus<br>**Olfactory bulb**: neg<br>**Lung**: one pos EC in one bronchiole<br>**BLN**: several pos macrophages/DC	-	-	-
**6 x 10^3 PFU**<br>[1]	**2**	**Nose**: several patches of and individual pos REC; some cell free viral Ag in mucus<br>**Olfactory bulb**: neg<br>**Trachea**: neg<br>**Lung**: neg	-	-	-
**6 x 10^3 PFU**<br>[2]	**2**	**Nose**: several patches of and individual pos REC and OEC; cell free viral Ag in mucus<br>**Olfactory bulb**: neg<br>**Trachea**: several patches of pos EC<br>**Lungs**: viral Ag covering luminal surface of bronchioles<br>**BLN**: neg	-	-	-
**6 x 10^3 PFU**<br>[3]	**2**	**Nose**: abundant patches of and individual pos REC; some cell free viral Ag in mucus<br>**Olfactory bulb**: neg<br>**Trachea**: neg<br>**Lung**: neg	+	+	+
**6 x 10^3 PFU**<br>[4]	**2**	**Nose**: large patches of and individual pos REC; some cell free viral Ag in mucus<br>**Olfactory bulb**: neg<br>**Trachea**: neg<br>**Lung**: neg	++	+	++
